# Resonant driving of a single photon emitter embedded in a mechanical oscillator

**DOI:** 10.1038/s41467-017-00097-3

**Published:** 2017-07-14

**Authors:** Mathieu Munsch, Andreas V. Kuhlmann, Davide Cadeddu, Jean-Michel Gérard, Julien Claudon, Martino Poggio, Richard J. Warburton

**Affiliations:** 1 Department of Physics, University of Basel, Klingelbergstrasse 82, CH-4056 Basel, Switzerland; 2 Univ. Grenoble Alpes, CEA, INAC, PHELIQS, “Nanophysique et semiconducteurs“ Group, F-38000 Grenoble, France

## Abstract

Coupling a microscopic mechanical resonator to a nanoscale quantum system enables control of the mechanical resonator via the quantum system and vice-versa. The coupling is usually achieved through functionalization of the mechanical resonator, but this results in additional mass and dissipation channels. An alternative is an intrinsic coupling based on strain. Here we employ a monolithic semiconductor system: the nanoscale quantum system is a semiconductor quantum dot (QD) located inside a nanowire. We demonstrate the resonant optical driving of the QD transition in such a structure. The noise spectrum of the resonance fluorescence signal, recorded in the single-photon counting regime, reveals a coupling to mechanical modes of different types. We measure a sensitivity to displacement of 65 fm/$\sqrt {{\rm{Hz}}}$ limited by charge noise in the device. Finally, we use thermal excitation of the different modes to determine the location of the QD within the trumpet, and calculate the contribution of the Brownian motion to the dephasing of the emitter.

## Abstract (web summary)

Resonant driving of a nanoscale quantum system coupled to a microscopic mechanical resonator may have uses in precision sensing and quantum information. The authors realize this by tailoring the geometry of a semiconductor nanowire embedding a quantum dot, detecting sub-picometre displacements.

## Introduction

Coupling a nanoscale quantum system to a microscopic mechanical resonator offers a new degree of freedom with potential applications in precision sensing and quantum information^1^. On the one hand, the mechanical resonator can be controlled via the quantum system, enabling ‘phonon lasing’^2, 3^ or cooling towards the mechanical ground state^2, 4^. On the other hand, the quantum system can be controlled via the mechanical system, offering the perspective of non-demolition readout via a precise measurement of the oscillator’s position^3^ and applications in precision sensing.

Mechanical resonators can be constructed on the micro- or nanoscale from various solid-state materials such as silicon nitride^5, 6^, silica^7^, silicon^8^, diamond^9^, GaAs^10^ and so on. Position readout of the mechanical resonator is usually carried out by incorporating the mechanical resonator in an optical cavity^11^. We pursue an alternative here, position readout by embedding a single photon emitter into the mechanical resonator itself.

GaAs is a natural choice of material for this endeavour. First, GaAs mechanical resonators are easy to make and have good mechanical properties^12, 13^. Second, a self-assembled quantum dot (QD) in GaAs represents an excellent single-photon emitter. At low temperature with resonant excitation, a QD is a fast, bright and pure source of single photons^14–19^, outperforming any other solid-state emitter. A crucial feature is that the QD transition frequency is sensitive to the strain induced by a deformation of the host material: there is an inherent coupling between the mechanics and the optical properties of the single-photon emitter^4^.

A coupling between a QD exciton (an electron–hole pair) and a mechanical oscillator was observed in two recent experiments^20, 21^. There, the readout signal resulted from the non-resonant excitation of the QD. This is not an ideal situation since the quality of the emitted photons is low (as inferred from a large spectral linewidth and low indistinguishability). The mechanical resonator was driven externally and shifts in the QD’s luminescence spectrum were demonstrated. While this non-resonant excitation scheme is sufficient for a first characterization of the coupling strength, it severely limits the potential of the device in sensing applications, and cannot be exploited to manipulate the mechanical oscillator.

We report here resonant optical driving of a high-quality QD embedded in a mechanical system. We use the resonance fluorescence (RF) signal to detect sub-picometre displacements of the mechanical modes at cryogenic temperature by measuring the fluctuations in the single-photon count rate, an amelioration in position sensitivity by four orders of magnitude compared to previous results^20, 21^. We use a Hanbury Brown–Twiss configuration to record the signal auto-correlation and reveal a pronounced coupling to high-frequency mechanical modes up to 50 MHz. As an application of the sensing capabilities of our device, we use the thermal excitation of a series of mechanical modes to determine the location of the QD within the nanowire. Finally we discuss the impact of the strain coupling on the coherence of the single-photon emitter. Our experiment demonstrates the use of an embedded single photon emitter for displacement read-out, and represents a first step towards adding and removing phonons to the mechanical oscillator via resonant optical driving of the emitter.

## Results

### Resonant spectroscopy of a QD coupled to a mechanical resonator

The mechanical system, a semiconductor nanowire with a conical taper, a ‘photonic trumpet’^22^, has been carefully selected to optimize both mechanical and optical properties simultaneously. The extra mass located at the end facet of the nanowire produces large strains in the ‘stem’ of the nanowire where the QD is located, resulting in large couplings between the QD and the mechanical resonator. The QD emits photons preferentially into the one-dimensional waveguide mode defined by the nanowire. The mode then expands adiabatically within the inverse taper, allowing very large couplings to a Gaussian mode in free-space^23^, resulting in very large photon extraction efficiencies. Importantly, we demonstrate here that the large and flat top facet, which neither clips nor depolarizes the excitation beam, allows for excellent suppression of the back-scattered light from the resonant laser. This solves an important challenge associated with the resonant spectroscopy of nano-sized (sub-wavelength) structures.

**Fig. 1:**
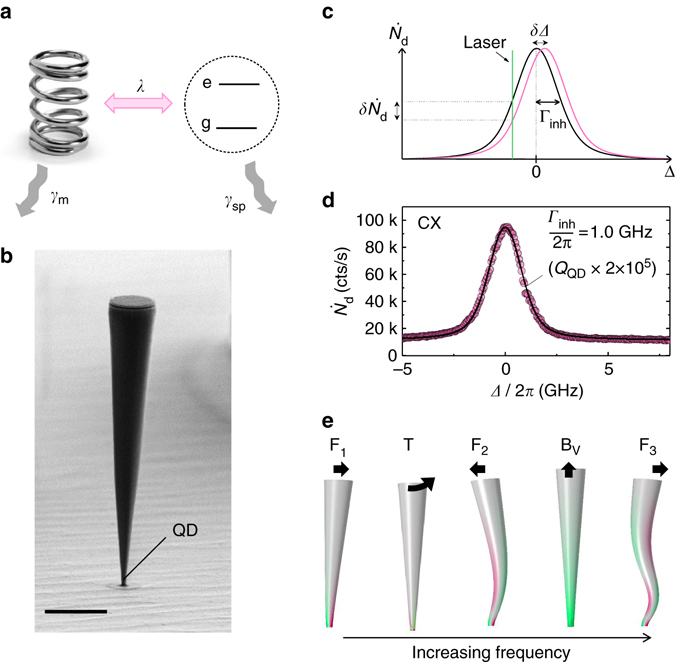
Quantum dot coupling to a nanowire mechanical resonator. **a** Sketch of the hybrid system: a mechanical oscillator is coupled to a two-level quantum system. The coupling rate *λ* (*pink*) competes with the dissipation rates of both components (*grey*): the intrinsic phonon relaxation rate *γ*_*m*_ and the spontaneous emission rate of a photon *γ*_sp_. **b** Practical realization: a quantum dot (QD) is embedded close to the bottom of a micrometre-sized mechanical resonator. The coupling originates from strain as the nanowire oscillates. The scale bar corresponds to 2 μm. **c** Effect of the coupling: a displacement *u* of the nanowire produces a shift *δΔ* in the QD frequency, modifying the detuning between the QD and the laser. *Γ*_inh_ is the linewidth of the QD, inhomogeneously broadened by spectral fluctuations. **d** Resonance fluorescence signal from the charged exciton (CX) as a function of laser detuning (fluorescence wavelength: 945.6 nm). ${{\it{\Omega}} _{\rm{R}}} \simeq {\gamma _{{\rm{sp}}}} = 1.1$ GHz. The fit uses a Voigt profile with a contribution to the linewidth of 0.45 GHz from the Lorentzian part, and 0.70 GHz from the Gaussian one. **e** The mechanical modes: F_1_, F_2_ and F_3_ correspond to the first, second- and third-order flexural modes; T to a torsional mode and *B*_v_ to a vertical breathing mode. The *colour map* represents the strain along the vertical axis within the trumpet (*green*: tensile, *pink*: compressive). The *black arrows* represent the displacement of the nanowire’s top facet

The QD–mechanical coupling manifests itself as a time-dependent frequency shift of the QD transition as the resonator oscillates. The frequency shift is determined by the strain coupling *λ* ^20, 21^ (Fig. 1a and c). This is described by the interaction Hamiltonian

1 $${\hat H_{{\rm{int}}}} = \frac{{\hbar \lambda }}{{{u_{{\rm{zpf}}}}}}\hat u\,{\hat \sigma _z},$$

where the Pauli operator ${\hat \sigma _z} = \frac{1}{2}\left( {\left| e \right\rangle \left\langle e \right| - \left| g \right\rangle \left\langle g \right|} \right)$ acts on the QD two-level system, $\hat u$ is the operator representing the nanowire’s displacement, *u*_zpf_ corresponds to the quantum zero-point fluctuations and $\lambda = \frac{{\partial \Delta }}{{\partial u}}{u_{{\rm{zpf}}}}$. To readout the displacement of the mechanical resonator, we drive the optical transition of an embedded QD with a linearly polarized narrow band laser and collect the scattered light in the orthogonal polarization^24^. By doing so we detect the RF from the QD and limit the amount of back-scattered laser light. A displacement *u* of the mechanical oscillator results in a detuning Δ of the QD with respect to the constant frequency laser and translates into a change $\delta {\dot N_{\rm{d}}}$ in the detected RF count rate (Fig. 1c). Assuming small optical detunings due to the mechanical oscillation,

2 $$\delta {\dot N_{\rm{d}}} = \frac{{\alpha \,\hbar \lambda }}{{{u_{{\rm{zpf}}}}}}u,$$

where $\alpha = \partial {\dot N_{\rm{d}}}{\rm{/}}\partial \Delta$ depends on the spectral profile of the emitter.

We focus on a 12-μm-long photonic trumpet, featuring a bottom diameter of 300 nm and a top facet diameter of 1.62 μm, Fig. 1b. The wire is clamped to a bottom gold-silica mirror via a flip-chip procedure^22^. The typical spectrum from a QD inside such a wire is shown in Fig. 1d. It is obtained from an excitation with two lasers: a very weak non-resonant laser is used to stabilize the QD’s charge environment^25^ while a second laser scans the QD transition. We observe a maximum in the RF as the second laser hits the QD resonance (*Δ* = 0), on top of a photoluminescence background associated with the non-resonant pump. The large top facet does not cause depolarization of the excitation beam, which enables an excellent suppression of the back-scattered light from the resonant laser (40 dB) and results in negligible background (signal-to-background ratio S:B = 125). To reach the best sensitivity of the QD to the mechanical motion, we have to maximize the count rate while maintaining a small linewidth. For this, we operate at the onset of power broadening (Rabi coupling ${{\it{\Omega}} _{\rm R} \simeq {\gamma }_{\rm sp}}$, with *γ*_sp_ = 1.1 GHz, the spontaneous emission rate of the QD). This results in a linewidth *Γ*_inh_/2*π* = 1.0 GHz (Fig. 1d) corresponding to an ‘optical quality factor’ *Q* = 2 × 10^5^.

### Detection of mechanical thermal motion

We now turn to the noise spectroscopy of the RF signal and determination of the strain coupling.

**Fig. 2:**
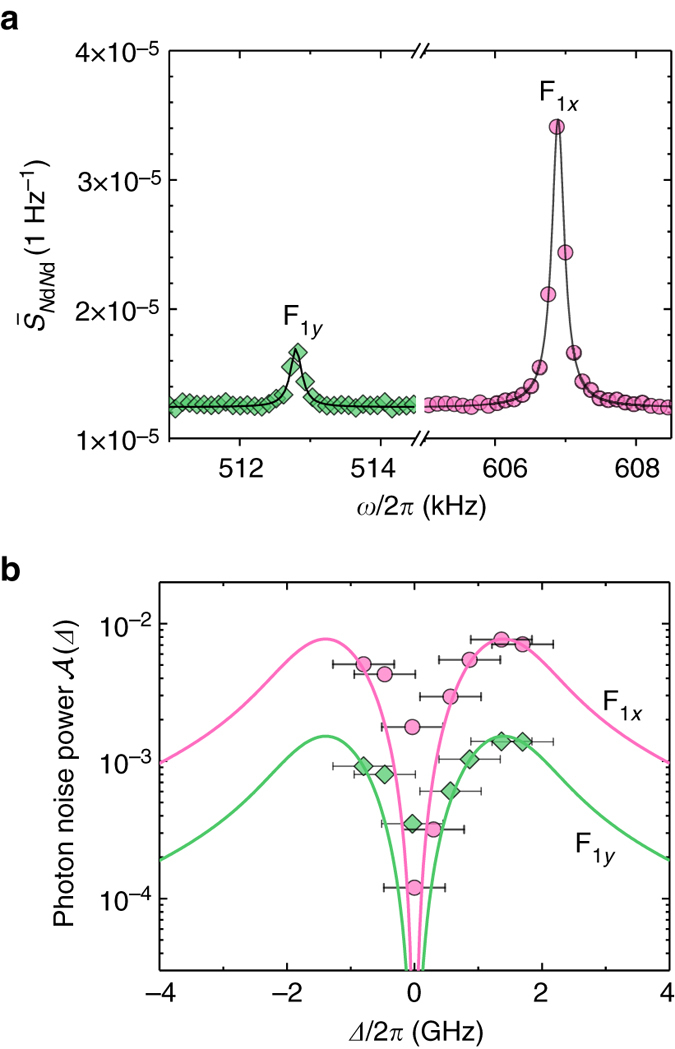
Brownian motion of the fundamental mechanical mode. **a** Photon noise power spectral density recorded at detuning *Δ* = *Γ*_inh_ (integration time 20 min). The resonances correspond to Brownian motion of the first-order flexural modes. The phonon relaxation rate for mode F_1*x*_ (F_1*y*_) is *γ*_m_/2*π* = 220 Hz (*γ*_m_/2*π* = 230 Hz). The background corresponds to the shot noise level. **b** Photon noise power ${\cal A}$ as a function of laser detuning for F_1*x*_ (pink circles) and F_1*y*_ (green diamonds). The solid lines are fits to the experimental data with *λ* as the only free parameter. The error bars correspond to the uncertainty on the quantum dot laser detuning over the 20 min measurement time

*Protocol 1.* One measurement technique to detect the mechanical motion is to record a time-trace of the QD RF at a fixed laser detuning Δ, Fig. 1c, and perform a Fourier analysis on the data^26^. Fig. 2a shows ${\bar S_{{{\rm{N}}_{\rm{d}}}{N_{\rm{d}}}}}$, the normalized noise power spectral density, computed from a 20 min time-trace recorded at *Δ* = *Γ*_inh_. The spectrum reveals two sharp resonances, labelled F_1*y*_ and F_1*x*_, at ${\omega _{{\rm{m}},{{\rm{F}}_{{{1y}}}}}}{\rm{/}}2\pi = 512.8$ kHz and ${\omega _{{\rm{m}},{{\rm{F}}_{{{1x}}}}}}{\rm{/}}2\pi = 607.9$ kHz, respectively. These resonances, which are absent in the bulk sample, correspond to the thermally driven mechanical resonances, i.e., the Brownian motion at 4 K. Specifically, we observe the two first-order flexural modes, whose degeneracy is lifted by a slightly anisotropic cross section.

The sensitivity of the measurement to the laser detuning is shown in Fig. 2b, where we plot the noise power associated with each mode, i.e., the area ${\cal A} = {\int} {{{\bar S}_{{{\rm{N}}_{\rm{d}}}{N_{\rm{d}}}}}\left( f \right)\,{\rm{d}}f}$ under each peak. We find from equation (2)

3 $${\cal A}({\it{\Delta}} ) = {\left( {\lambda \frac{{{u_{{\rm{th}}}}}}{{{u_{{\rm{zpf}}}}}}\frac{{\alpha ({\it{\Delta}} )}}{{\left\langle {{{\dot N}_{\rm d}}({\it{\Delta}} )} \right\rangle }}} \right)}^{2},$$

where *u*_th_ and *u*_zpf_ correspond to the thermal and zero-point fluctuations, respectively. These quantities depend in particular on the characteristics of the mechanical resonator, namely its mode frequency and motional mass. The former is obtained from our measurement, while the latter is determined through a finite element simulation of the resonator. *u*_th_ depends on the mode temperature, taken as 4 K assuming thermalization of the oscillator to the He bath (see section II 3 and Methods). For the first flexural mode we find *u*_zpf_ = 2.3 × 10^−14^ m and *u*_th_ = 1.2 × 10^−11^ m. The detuning dependence of $\left\langle {{{\dot N}_{\rm d}}({\it{\Delta}} )} \right\rangle$ and *α*(*Δ*) are obtained from a fit of the RF spectrum in Fig. 1c, so that equation (3) eventually only depends on the strain coupling *λ*. Using ${\lambda _{{{\rm{F}}_{{{1x}}}}}}{\rm{/}}2\pi = 280$ kHz and ${\lambda _{{{\rm{F}}_{{{1y}}}}}}{\rm{/}}2\pi = 55$ kHz, we find good agreement with the experimental data. The bandwidth of this measurement protocol is limited by the detector’s dead-time. In our case, this means a cutoff at a frequency of 10 MHz.

**Fig. 3:**
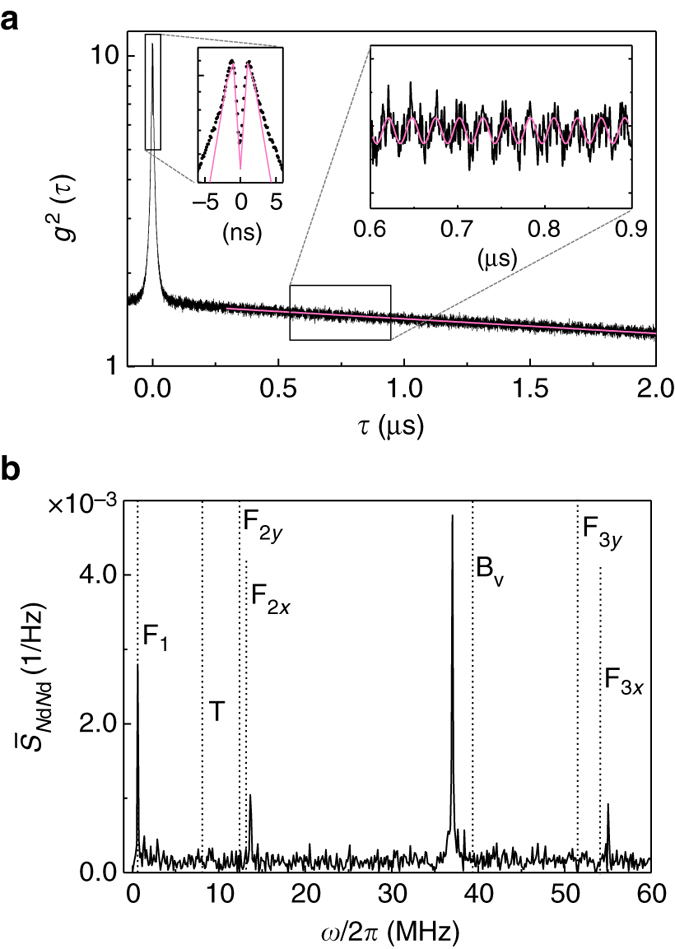
Quantum dot-mechanical coupling in the photon counting regime. **a** Auto-correlation function recorded at ${\it{\Delta}} \simeq {{\it{\Gamma}} _{{\rm{inh}}}}$ (the entire data extends over 8 μs). The oscillation in the right inset results from the mechanically induced modulation in count rate (the fit is a sine with a period of 27 ns). The fit in the left inset (*pink solid line* corresponds to a perfect single-photon emitter subject to blinking (*Ω*_R_ = *γ*_sp_) and includes the timing jitter of the detectors (500 ps). **b** In *black*, the noise power spectral density obtained from the Fourier transform of the auto-correlation measurement. The black dotted lines correspond to the resonance frequencies obtained from a simulation

*Protocol 2.* To probe the strain coupling in the MHz range, we perform an auto-correlation measurement of the RF signal with two detectors in a Hanbury Brown–Twiss configuration^27^. Figure 3a shows the result from a 70 min measurement recorded at *Δ* = *Γ*_inh_. The dip at zero delay is the signature of single-photon emission from the QD. Its moderate depth is a consequence of the timing jitter of the detectors (note that the dip is narrowed by the unresolved Rabi oscillations induced by the amplitude of the drive, *Ω*_R_ ~ γ_sp_). The bunching peak at short delays (*τ* < 100 ns) is related to a blinking in the QD emission^28^. The peak value increases as the on:off ratio in the QD emission diminishes; the decay time of the bunching is a measurement of the correlation time of charge fluctuations in the QD environment^29^. In addition to these features, we observe a small oscillation, with a period of  ~ 25 ns, which runs over the entire 8 μs time-span of the experiment. This oscillation corresponds to the signature of the strain coupling in the photon counting regime.

A Fourier analysis of the intensity correlation data at *τ* > 0.25 μs is shown in Fig. 3. The spectrum reveals a whole series of resonances corresponding to different modes of the mechanical resonator. In particular, the lower-frequency mode corresponds to the first-order flexural mode (F_1_) already evidenced in Fig. 2, and the pronounced peak at 37 MHz corresponds to the vertical breathing mode (*B*_v_) immediately visible in the time-dependent data. Complete mode assignment is obtained from a numerical simulation. With fine adjustment of the trumpet’s dimensions (see Methods) we are able to reproduce the spectrum within a maximum error of 7.5% in the exact frequency (vertical dotted lines in Fig. 3b). In particular, we find that an ellipticity in the base diameter of 20% accounts for the splitting observed in Fig. 2b, in good agreement with earlier work^20^. Quite remarkably, we observe a pronounced amplitude for *B*_v_ despite the smaller phonon population associated with this high-frequency mode (*u*_th_ = 1.4 × 10^−13^ m). This is the consequence of the large strain field associated with this specific mode. Quantitatively, this translates into a coupling ${\lambda _{{{\rm{B}}_{\rm{v}}}}}{\rm{/}}2\pi = 3.6$ MHz, much larger than the values obtained for the mode F_1_. The dissipation rate of B_v_ also significantly increases, reaching ${{\rm{\gamma }}_{{\rm{m}},{{\rm{B}}_{\rm{v}}}}}{\rm{/}}2\pi = 0.14$ MHz.

The measured noise spectrum may be translated into an equivalent displacement noise spectrum. For F_1_, this results in a sensitivity to the displacement of the top facet of 2.6 × 10^−13^ m Hz^−1/2^ ($\sqrt {{S_{uu}}}$ = 6.5 × 10^−14^ m Hz^−1/2^ for B_v_). At present, this value is limited by charge noise in the device. For our system, this nevertheless represents reading displacement amplitudes equal to the zero-point fluctuations in just 70 s.

### Quantum dot position determination

As a first application of the sensing capabilities of our device, we use the QD’s sensitivity to the local strain to determine the exact location of the QD inside the photonic wire. This is a non-trivial task, important, e.g., for the coupling of two emitters via optical modes^30^. To do so, we use the thermal excitation of a series of modes. The idea is that each mode is driven equally by the thermal noise and produces a specific strain at a given location in the wire. By comparing the relative amplitude of the measured resonances, it is possible to extract the position of the QD inside the wire.

**Fig. 4:**
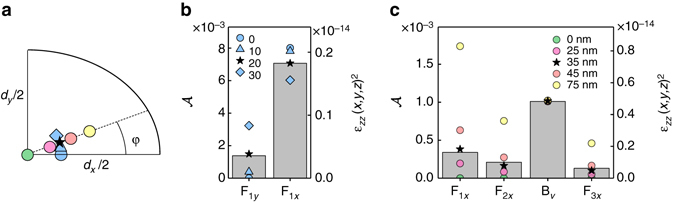
Determination of the QD position inside the nanowire. **a** One quadrant of the QD plane (*x* and *y* correspond to the long and short axis of the ellipse, respectively). *Coloured symbols* correspond to various QD positions which result in the spectral signatures in **b** and **c**. The *black star* corresponds to the position of QD1. **b** *Grey bars*, *left axis*: experimental data obtained in protocol 1. *Symbols, right axis*: simulated strain along the *z* axis for four different angles (in degrees) and a fixed distance (*r* = 35 nm) to the centre (a different distance results in a linear scaling of the right axis). **c** Comparison between experiment (protocol 2) and simulated strain along the *z* axis for various QD distances to the centre (*ϕ* = 20°)

The procedure goes as follows. We simulate the strain corresponding to a unit displacement of the top facet for a QD at a fixed distance of 110 nm from the base. (In our case, the situation is simplified by the fact that we already know the precise location of the QD layer in the *z*-direction from the growth.) We then compute the exact displacement associated with each mode, which corresponds here to the average displacement resulting from the Brownian motion at 4 K. From this proportionality factor, we obtain the strain corresponding to the Brownian motion. Table 1 shows the results for a QD located on the *x* axis, at a 35 nm distance from the centre (blue circle in Fig. 2a). We observe that for all modes except T, the strain ${\epsilon _{zz}}$ in the vertical direction dominates over the other components. In fact we find that ${\varepsilon _{xx}} \approx {\varepsilon _{yy}} \approx - \nu \,{\varepsilon _{zz}}$, where *ν* = 0.31 is the Poisson ratio: to a good approximation, the QD experiences a uniaxial stress along the *z*-direction^23, 30^. This results in ${\bar S_{NN}} \propto \epsilon _{zz}^2$. To determine the QD’s position, we first vary the in-plane angle *ϕ* for a fixed distance of the QD to the centre, and calculate the relative amplitude of the first-order flexural modes(*F*_1*x*_ and *F*_1*y*_). This is shown in Fig. 4b, where we plot the results for four different values of *ϕ* and scale it to the experimental result. In a second step, we vary the radial position until we get a good agreement with the amplitudes of the entire series of higher-order modes, Fig. 4c. This technique allows for an accurate positioning of the QD, modulo a symmetry versus the *x* and *y* axes (this experiment does not resolve positive and negative coordinates). We find that QD1 is located 35 nm away from the axis of the trumpet, with an angle *ϕ* = 20° (black star in Fig. 4).

**Table 1:** Simulated strain for a QD on the *x* axis for the different mechanical modes

Mode	*ω*_m_ (MHz)	*u*_th_ (pm)	${\boldsymbol{\varepsilon} _{zz}}$	${\boldsymbol{\varepsilon} _{yy}}$	${\boldsymbol{\varepsilon} _{xx}}$
F_1*x*_	0.61	12	4.5 × 10^−8^	−1.3 × 10^−8^	−1.5 × 10^−8^
B_1_	8.2	1.2	1.4 × 10^−11^	−1.1 × 10^−11^	−7.5 × 10^−12^
F_2*x*_	13.4	0.45	3.0 × 10^−8^	−8.1 × 10^−9^	−9.7 × 10^−9^
B_2_	40.0	0.14	7.0 × 10^−8^	−2.1 × 10^−8^	−1.9 × 10^−8^
F_3*x*_	55.0	0.10	2.3 × 10^−8^	−6.3 × 10^−9^	−7.6 × 10^−9^

The QD is located 35 nm away from the centre along the *x*-axis. Details of the geometry of the nanowire are given in Section IV 5. The strain values are given for a displacement of the top facet equal to the time-averaged displacement *u*_th_ at *T* = 4.2 K

Some comments are in order. First, the assumption that the system is at bath temperature is tested *in situ*: the QD is an excellent thermometer and we observe, in particular, no laser-induced heating. Second, a related idea was introduced recently in ref. ^30^. In this work, the authors measure a shift in the QD energy as they drive the motion of the wire (addressing F_1*x*_ and F_1*y*_ successively). From the calibration of the induced displacement, and previous knowledge of QD response to strain, it is possible to determine the position of the QD. In the present situation, the use of a series of modes, all driven by thermal noise, allows for a more direct analysis with no need for an external calibration of the QD sensitivity to strain. (Note that B_v_, in particular, produces a constant strain for all QDs in a given cross section plane of the wire and is very conveniently used as a reference point.) Furthermore, our technique requires in principle no previous knowledge of the location of the QD within the *z* axis and could thus be used for a full three-dimensional mapping of the QD position.

## Discussion

A quantum emitter in a nanowire has potential for various quantum applications. On the one hand, dielectric nanowires are used as waveguides to realize high-fidelity single-photon sources in quantum optics^14, 15, 31, 32^. In this context, the strain coupling represents an additional dephasing channel with potential impact on the photon indistinguishability. Indeed, the coupling to mechanical modes introduces noise which leads to a broadening of the QD transitions for integration times longer than the mechanical oscillation period, i.e., the coupling reduces the indistinguishability for photons separated by times larger than the oscillation period. The amplitude of the energy shift induced by the Brownian fluctuations of the nanowire is given by

4 $$\delta {{\it{\Delta}} _{{\rm th}}} = \lambda \frac{{{u_{{\rm th}}}}}{{{u_{{\rm zpf}}}}}.$$

In the present situation, we find $\delta {{\it{\Delta}} _{{\rm{th,}}{{\rm{F}}_{{{1x}}}}}}{\rm{/}}2\pi = 0.15$ GHz and $\delta {{\it{\Delta}} _{{\rm{th,}}{{\rm{B}}_{\rm{v}}}}}{\rm{/}}2\pi = 0.24$ GHz for F_1*x*_ and B_v_, respectively. For our device, this represents a slow $( w_m \ll \gamma_{\rm sp})$ and negligible dephasing $\left( {\delta {{\it{\Delta}} _{{\rm{th}}}} \ll {{\it{\Gamma}} _{{\rm{inh}}}}} \right)$. The values, however, compare with the minimum dephasing rate achievable (0.17 GHz), set by the spontaneous decay rate. More generally, we stress that such a coupling cannot be turned off and is present in the large majority of QD devices involving micro-fabrication, unless a specific engineering is used^33, 34^. Recent results have shown that QDs in micropillars could be used for the generation of close to indistinguishable single photons with high collection efficiency^18, 19, 35^. Our simulations indicate that while the QD-mechanical coupling is strongly suppressed for flexural modes, the vertical breathing mode still leads to sizable dephasing (few tens of MHz for a 2 μm high pillar with 1.5 μm diameter) on very short timescales (in this example the resonance frequency for B_v_ is 0.5 GHz). These results reveal the importance of considering the mechanical properties of any device designed for perfect single-photon emission.

On the other hand, the large strain couplings suggest further applications using the nanowire as a quantum resonator. To this end, B_v_ stands out as a very interesting mode, with a cooperativity ${{\cal C} = {\lambda}^2{\rm{/}}{\gamma}_{\rm sp}{\gamma}_{\rm{m}}} = 0.6$. In the present experiment, the number of phonons in the mode is still high $\left( {{n_{{\rm{th,B2}}}} \simeq 2200} \right)$. Cooling the system down to 20 mK would result in ${n_{{\rm{th}}}} \simeq 11$, corresponding to a quantum cooperativity ${{\cal C}_Q} = {\cal C}{\rm{/}}{n_{{\rm{th}}}} = 0.06$. While this does not yet allow for coherent exchange between the QD and the mechanical resonator, the resonant excitation demonstrated here also opens the possibility of using the embedded QD to further cool the mechanical oscillator^4^. To that end, smaller structures with larger mechanical frequencies leading to the resolved side-band regime will facilitate operation. This does not represent a technological obstacle, meaning that such experiments are within reach. We emphasize that the present device could already be used to generate coherent mechanical vibrations from the QD excitation, as suggested by Auffèves and Richard^3^ in a recent proposal. In this case, only the mechanical damping is important: the number of coherent phonons created through the optical driving of the two-level system depends on (*λ*/*γ*_m_)^2^. For both F_1_ and B_v_, $\lambda \gg {\gamma _{\rm m}}$. For F_1_, applying this scheme to the present device, we predict a QD-induced r.m.s. displacement as large as 80 pm, while the termal motion at 4 K represents an r.m.s. displacement of 12 pm.

Finally, we speculate that the Heisenberg limit in displacement sensitivity is achieved for an emitter driven close to but below saturation provided that the emitter has a transform-limited linewidth and that the photons are collected and detected with perfect efficiency. Given the recent progress in QD micropillars^18^, this limit is within experimental reach.

## Methods

### Resonance fluorescence

The challenge with resonant spectroscopy is to distinguish between the fluorescence signal and the back-scattered laser light. For this we use a dark-field microscope based on cross-polarized excitation and detection. This technique ensures extinction ratios as high as 10^7^ upon reflection on a flat surface^24^. The situation is however more complex when the QD environment is processed below the micrometre scale: a small object in the focus of the incident laser causes depolarization of the reflected beam and prevents efficient rejection. To reconcile the nanoscale engineering of the QD environment with the need for a flat top facet, we use a tapered nanowire, the ‘photonic trumpet’. A layer of QDs is embedded at the base of the structure where the lateral dimensions (between 200 and 260 nm) force the emission into the fundamental photonic mode propagating in the vertical direction^36^. The subsequent tapered section results in an adiabatic deconfinement of the mode and limits diffraction losses at the top facet^22^. For the photonic trumpet studied in this paper (bottom diameter 300 nm, top diameter 1.62 μm), we achieve a laser suppression >40 dB over a 29 GHz frequency span with fixed settings of the polarizers. This results in a signal to noise ratio *S*:*N* = 125 at a driving amplitude *Ω*= *γ*_sp_. To obtain a spectrum, we sweep the laser frequency with a constant rate. The resulting resolution is 85 MHz, with an integration time of 0.2 s per point.

### Photon collection efficiency

At saturation, we detect a maximum RF count-rate of 0.83 MHz. Together with the measured *γ*_sp_ = 1.1 GHz, this yields $\epsilon$ = 0.16% for the overall efficiency. Taking the response of the detectors (21%), the transmission of our setup (7%) and blinking phenomena (QD ‘on’ time 10%) into account, we calculate the photon collection efficiency at the first lens (NA = 0.8) which amounts to 36%. This is about a factor two below previously reported measurement on similar structures^22^. We attribute the difference to the larger diameter of the nanowire at the QD position and to the position of the QD away from the axis (a necessary condition to observe the response from the first order flexural mode, see also section II 3): all these factors reduce the coupling to the fundamental guided mode.

### Normalized noise power spectral density

In the first protocol, we record a time trace ${\dot N_{\rm d}}(t)$ of the detected count-rate and compute the normalized noise spectrum

5 $${\bar S_{NN}}(f) = 2\,{\rm{FT}}{\left[ {\frac{{{{\dot N}_{\rm d}}(t)}}{{\left\langle {{{\dot N}_{\rm d}}(t)} \right\rangle }}} \right]^2}\frac{{t_{{\rm{bin}}}^2}}{T},$$

where *t*_bin_ is a post-selected binning time, *T* is the total integration time and $\left\langle {{{\dot N}_{\rm d}}(t)} \right\rangle$ is the average number of counts per bin. In the second protocol, we record the auto-correlation function ${g^{(2)}}(\tau ) = \frac{{\left\langle {{{\dot N}_{\rm d}}(t){{\dot N}_{\rm d}}\left( {t + \tau } \right)} \right\rangle }}{{{{\dot N}_{\rm d}}{{(t)}^2}}}$ of the signal whose Fourier transform directly gives the normalized noise spectrum. Both protocols are connected by the Wiener–Khinchin theorem, but their bandwidths differ. In the first case, the spectrum is limited by the dead-time of the detector (100 ns). In the second case, the bandwidth cutoff frequency is pushed further and determined by the jitter of the detectors (40 ps).

### Evaluation of the coupling strength

We determine the strength of the strain coupling from the assumption that the oscillator is fully thermalized with the surrounding He bath. Using the equipartition theorem,

6 $$\left\langle {{u^2}} \right\rangle = \frac{{{k_{\rm{B}}}T}}{{{m_{{\rm{eff}}}}\omega _{\rm m}^2}} = u_{{\rm{th}}}^{\rm{2}}.$$

In addition, we have from equation (2)

7 $$\left\langle {{u^2}} \right\rangle = \frac{{u_{{\rm{zpf}}}^2}}{{{\alpha ^2}\,{\lambda ^2}}}\left\langle {\delta \dot N_{\rm{d}}^2} \right\rangle ,$$

with *α* the derivative of the QD spectrum. We introduce the normalized photon noise spectrum ${\bar S_{NN}}$,

8 $$\left\langle {\delta \dot N_{\rm{d}}^2} \right\rangle = {\left\langle {{{\dot N}_{\rm{d}}}} \right\rangle ^2}{\int} {{{\bar S}_{NN}}} \left( f \right){\rm d}f,$$

where ${\int} {{{\bar S}_{NN}}(f){\rm{d}}f = {\cal A}}$ is the photon noise power. We obtain ${\cal A}$ for each mode from the area below the corresponding peak in the power noise spectrum (Fig. 2a). This yields the equation to describe the experimental data in Fig. 2b

9 $${\cal A}({\it{\Delta}} ) = {\left( {\lambda \frac{{{u_{{\rm{th}}}}}}{{{u_{{\rm{zpf}}}}}}\frac{{\alpha ({\it{\Delta}} )}}{{\left\langle {{{\dot N}_{\rm d}}({\it{\Delta}} )} \right\rangle }}} \right)}^{\!\!2},$$

which only depends on *λ*. We point out that the approximation is not only realistic (our sample is located in He exchange gas) but also verified using the QD itself, which represents a sensitive thermometer. In particular, we observe no shift of the resonance as we crank up the power of resonant and non-resonant lasers up to saturation.

### Numerical analysis

To confirm the origin of the resonances in our noise spectrum, we calculate the mechanical eigen-frequencies of the resonator using a commercial finite-element analysis software (Comsol). We simulate a 12 μm long GaAs wire, with a bottom diameter of approximately 300 nm and a tapering angle of *θ* = 3°. In order to adjust the mechanical frequencies to the experimental values, we allow a 5% variation in the length of the wire (arising from flux inhomogeneities over the wafer surface in the molecular beam epitaxy chambre). To account for the observed splitting of the first flexural mode, we also introduce a small asymmetry in the QD plane. In practice, the nanowire has a round top diameter but an elliptic base, the consequence of a slightly anisotropic etching process (Δ*θ* = *θ*_*x*_ − *θ*_*y*_). Figure 4 shows the results for a 11.4-μm-long wire, with a top diameter of 1.62 m and a bottom section with minor axis *d*_*y*_ = 260 nm and major axis *d*_*x*_ = 320 nm (Δ*θ* = 0.15°, corresponding to the estimation of the anisotropy in the etching process^20^).

### Measurement on a second QD

**Fig. 5:**
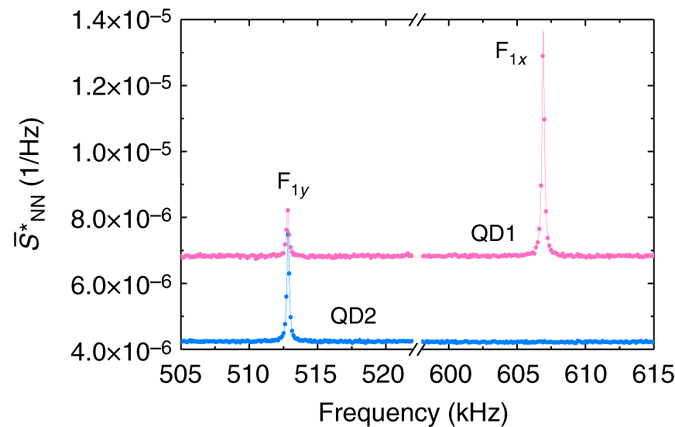
QD noise spectrum for two different QDs. The shot noise has been subtracted: $\bar S_{NN}^* = {\bar S_{NN}} - \bar S_{NN}^{{\rm{shot}}}$. QD1 corresponds to the QD studied in the main paper. QD2 belongs to the same nanowire and its noise spectrum is recorded under similar conditions: ${\Omega _{\rm R}} \simeq {\gamma _{{\rm{sp}}}}$, Δ = Γ, integration time of 20 min

We present results from the noise spectroscopy on a second QD in the same nanowire (QD2). The result is shown in Fig. 5, where we have removed the contribution from shot noise^26^. The different spectral signature is attributed to a different location of QD2 in the nanowire. In particular, the absence of a resonance at 607 kHz means that QD2 is located on the neutral (zero-strain) axis of F_1*x*_. We point out that amplitude of the peak is not a direct measurement of the relative coupling strength, since the QD linewidths may be different. In the present case, QD2 has a larger full-width at half-maximum (not shown) which lowers its sensitivity. The reduced noise floor is associated to this lower sensitivity, possibly combined with a less noisy charge environment around QD2.

### Quantum dot strain and optical frequency

We finally evaluate the expected QD frequency shift from the calculated strain. Neglecting confinement effects, this reads $\hbar \delta = a{\varepsilon _h} + \frac{b}{2}{\varepsilon _{{\rm{sh}}}}$, where ${\varepsilon _h} = {\varepsilon _{xx}} + {\varepsilon _{yy}} + {\varepsilon _{zz}}$ and ${\varepsilon _{{\rm{sh}}}} = 2{\varepsilon _{zz}} - {\varepsilon _{xx}} - {\varepsilon _{yy}}$ correspond to the hydrostatic and shear strains respectively, and *a* and *b* are material-dependent deformation potentials^23^. Including the effect of both tensile and compressive strain, this result in a mechanically induced dephasing *Δ*_th_ = 2*δ*. (Note that in equation (4) this factor 2 is included in the definition of *λ*.) Assuming the QD is mainly composed of GaAs^37^, *a* = −8.33 eV and *b* = −2.0 eV^23^, we find ${\it{\Delta}} _{{\rm{th}}}^{{\rm{F}}1{\rm{x}}}{\rm{/}}2\pi = 0.16$ GHz and ${\it{\Delta}} _{{\rm{th}}}^{{\rm{B}}2}{\rm{/}}2\pi = 0.20$ GHz, in excellent agreement with the results from Section III.

### Data availability

Data available on request from the authors.
